# The Role of Tumor Necrosis Factor-Alpha in the Pathogenesis and Treatment of Nonalcoholic Fatty Liver Disease

**DOI:** 10.1007/s13679-023-00519-y

**Published:** 2023-07-05

**Authors:** Ilias D. Vachliotis, Stergios A. Polyzos

**Affiliations:** 1 First Department of Pharmacology, Medical School, Aristotle University of Thessaloniki, 54124 Thessaloniki, Greece; 2 Second Department of Internal Medicine, 424 General Military Hospital, Thessaloniki, Greece

**Keywords:** Nonalcoholic fatty liver disease, Nonalcoholic steatohepatitis, Tumor necrosis factor-α, Inflammation, Treatment

## Abstract

**Purpose of Review:**

To summarize experimental and clinical evidence on the association between tumor necrosis factor-α (TNF-α) and nonalcoholic fatty liver disease (NAFLD) and discuss potential treatment considerations.

**Recent Findings:**

Experimental evidence suggests that TNF-α is a cytokine with a critical role in the pathogenesis of NAFLD. Although, the production of TNF-α may be an early event during the course of nonalcoholic fatty liver (NAFL), TNF-α may play a more substantial role in the pathogenesis of nonalcoholic steatohepatitis (NASH) and NAFLD-associated fibrosis. Moreover, TNF-α may potentiate hepatic insulin resistance, thus interconnecting inflammatory with metabolic signals and possibly contributing to the development of NAFLD-related comorbidities, including cardiovascular disease, hepatocellular carcinoma, and extra-hepatic malignancies. In clinical terms, TNF-α is probably associated with the severity of NAFLD; circulating TNF-α gradually increases from controls to patients with NAFL, and then, to patients with NASH. Given this potential association, various therapeutic interventions (obeticholic acid, peroxisome proliferator-activated receptors, sodium-glucose co-transporter 2 inhibitors, glucagon-like peptide-1 receptor agonists, probiotics, synbiotics, rifaximin, vitamin E, pentoxifylline, ursodeoxycholic acid, fibroblast growth factor-21, n-3 polyunsaturated fatty acids, statins, angiotensin receptor blockers) have been evaluated for their effect on TNF-α and NAFLD. Interestingly, anti-TNF biologics have shown favorable metabolic and hepatic effects, which may open a possible therapeutic window for the management of advanced NAFLD.

**Summary:**

The potential key pathogenic role of TNF-α in NAFLD warrants further investigation and may have important diagnostic and therapeutic implications.

## Introduction 

Nonalcoholic fatty liver disease (NAFLD) is a highly prevalent disease affecting approximately 30% of the general population; the prevalence is much higher in certain populations, such as patients with type 2 diabetes mellitus (T2DM) and obesity, reaching 60% and 90%, respectively [1]. Although NAFLD has become the most common chronic liver disease worldwide, it remains largely underdiagnosed, whereas the absence of any approved pharmacological therapy results in increased health and socioeconomic burden [2]. NAFLD does not represent a single entity, but it encompasses distinct phenotypes: nonalcoholic fatty liver (NAFL, simple hepatic steatosis), nonalcoholic steatohepatitis (NASH), in which steatosis is combined with inflammation, degeneration, and various degrees of hepatic fibrosis, and finally, NAFLD-related cirrhosis, either compensated or decompensated, and hepatocellular carcinoma (HCC) in a subset of patients [3].

NASH and hepatic fibrosis are associated with increased risk of hepatic complications, including cirrhosis and HCC, as well as extra-hepatic complications, including cardiovascular disease (CVD), chronic kidney disease, and extrahepatic malignancies [3]. NASH is characterized by high concentrations of multiple cytokines, including tumor necrosis factor-α (TNF-α) and interleukin (IL)-6, which may play key roles in disease pathogenesis [4–6]. TNF-α has been proposed to orchestrate an inflammatory process that extends beyond the liver [6]; however, although TNF-α seems to participate in the pathogenesis of NASH, its exact role has not been fully elucidated and warrants further investigation.

This review, first, focuses on the possible pathogenic role of TNF-α in NAFLD; second, summarizes clinical evidence on the association between TNF-α and NAFLD; and third, discusses potential treatment considerations derived from this association.

## TNF-α and Pathogenesis of NAFLD

### TNF-α in NAFLD: Origin and Biological Functions

NAFLD is characterized by a state of chronic, low-grade hepatic and systemic inflammation affecting multiple organs beyond the liver [6]. TNF-α is regarded as a key cytokine in this process, critically implicated in the pathogenesis of NAFLD. In NAFLD, TNF-α is not only produced in the liver by the resident hepatic cells, Kupffer cells (KCs), but also by the immune cells infiltrating the liver in the presence of steatosis [7]. Dysfunctional visceral adipose tissue also contributes to the production and secretion of TNF-α, mainly produced by immune cells infiltrating the adipose tissue, when it is expanded (e.g., in obesity); TNF-α of extra-hepatic origin is delivered to the liver via systemic circulation, along with other cytokines and adipokines, which may also affect the development and progression of NAFLD [7].

Obesity may affect the liver, at least partly, through the secretion of adipokines (e.g., leptin, adiponectin, resistin, visfatin), which are produced mainly but not exclusively by the adipocytes, and cytokines (e.g., TNF-α, IL-1, IL-6), which are produced mainly by the immune cells infiltrating the adipose tissue when it expands; there is an ever continuing and dynamic interplay among various adipokines and cytokines, exhibiting synergistic or antagonistic action [6, 8]. As for example, leptin and resistin were shown to upregulate the expression of TNF-α in the liver, thus contributing to the onset of NAFLD and to its progression to NASH [9, 10]. In contrast, adiponectin normally downregulates TNF-α expression in the liver, but its levels are low in obesity, thus TNF-α production is enhanced, with subsequent effects in the liver [5]. The above considering, circulating TNF-α derives from various sources, including the adipose tissue, and may affect distant tissues, including the liver.

In the liver, KCs respond to two main types of stimuli: the intrahepatic danger-associated molecular patterns (DAMPs), which are released by the lipid-infiltrated and damaged hepatocytes, and the gut-derived bacterial antigens, also known as pathogen-associated molecular patterns (PAMPs), which translocate from the intestine to the liver due to an impaired intestinal epithelial barrier [11]. Both DAMPs and PAMPs bind Toll-like receptors (TLRs) on the surface of KCs and activate the intracellular pathway of nuclear factor-kappa B (NF-κB), which is the key signaling pathway for the transcription of TNF-α, as well as other cytokines and chemokines [12]. Notably, hepatocytes that are stressed by fat accumulation (lipotoxicity) also contribute to TNF-α production via the interaction between DAMPs/PAMPs and TLRs, albeit to a lesser extent [11].

Once released, TNF-α manifests its biological functions by binding its cognate receptors, TNF receptor 1 (TNFR1) and TNF receptor 2 (TNFR2), which are expressed on the cell membrane [13]. Binding either TNFR1, which is ubiquitously expressed, or TNFR2, whose expression is restricted to immune and endothelial cells, TNF-α initiates two important downstream signaling pathways, i.e., the c-Jun N-terminal kinase (JNK) and NF-κB pathways [13]. Through these intracellular pathways, TNF-α induces the transcription of target genes involved in inflammation, cell proliferation, and survival [14]. Besides these cellular responses, TNF-α also induces cell death, which is only mediated by TNFR1 but not TNFR2. TNFR1 contains a death domain, which enables the assembly of a death-inducing protein complex that sufficiently mediates cell death [14].

### Contribution of TNF-α to NAFL, NASH, and NAFLD-Related Fibrosis

Although the production of TNF-α may be an early event in NAFL, potentially enhancing the severity of hepatic steatosis, NAFL is strongly associated with insulin resistance (IR), which is considered a major pathogenic driver of lipid accumulation in the liver [7]. As mentioned above, lipotoxicity is a main trigger for the production of TNF-α in the hepatocytes [15]; nevertheless, TNF-α may also influence the development of NAFL by modulating hepatic lipid metabolism. Indeed, transgenic mouse models of NAFLD lacking TNF-α receptors (TNFR − / −) were protected from severe hepatic fat accumulation compared with those with intact TNF-α signaling [16–18]. Moreover, treatment of NAFLD mice with anti-TNF antibody [19] or selective anti-TNFR1 antibody [20] attenuated hepatic steatosis. Of note, TNF-α was proposed as a potential positive regulator of the mammalian target of rapamycin (mTOR) complex-1 pathway in the hepatocytes, thus inducing the expression of the sterol regulatory element-binding protein (SREBP)-1c, a key transcription factor of *de novo* lipogenesis; it is highlighted that insulin is also a major regulator of the mTOR pathway [20]. An effective mTOR pathway is essential for both the expression and activation of SREBP-1c and subsequent upregulation of target lipogenic enzymes, which facilitates the intra-hepatic conversion of carbohydrates to free fatty acids, thus increasing lipid accumulation in the hepatocytes.

Apart from its possible role in the pathogenesis of NAFL, TNF-α may play a more substantial role in the pathogenesis of NASH and NAFLD-associated fibrosis [4]. In NASH, TNF-α provokes the release of a variety of pro-inflammatory mediators, including, but not limited to IL-1β, IL-6, IL-18, monocyte chemoattractant protein (MCP)-1, C–C motif chemokine 5 (CCL5), resulting in a massive activation of the immune response [21]. In addition, TNF-α induces the expression of adhesion molecules (e.g., intracellular adhesion molecule-1 (ICAM-1)) on the surface of the endothelial cells of liver vessels, which facilitates the migration of monocytes, neutrophils, and lymphocytes from the bloodstream into the liver, which, in turn, may produce further TNF-α, thus creating a vicious cycle [21]. As a result, TNF-α fuels a prolonged and sustained liver inflammation through the activation of inflammatory mediators, as well as migration and activation of immune cells.

Hepatocellular death, another important feature distinguishing NASH from NAFL, is reciprocally connected with inflammation, as both processes may positively regulate each other [22]. In the hepatocytes, TNF-α promotes two separate death mechanisms: apoptosis and necroptosis, regulated mainly through TNFR1 signaling [13]. Apoptosis is a caspase-dependent programmed death pathway, whereas necroptosis is another form of programmed cell death that is caspase-independent and is mediated by the receptor-interacting protein (RIP)1, RIP3, and mixed lineage kinase domain-like protein (MLKL) [13]. While apoptosis is considered silent and immunologically inert, necroptosis activates the inflammatory cascade, thus the immune response [23]. However, TNF-α/TNFR interaction has been regarded as a less potent inducer of cell death compared with other mediators of death signals, such as Fas ligand (FasL) and TNF-related apoptosis-inducing ligand (TRAIL), because its pro-apoptotic effect may be counteracted by its cytoprotective effect and its involvement in cell survival mediated by NF-κB [24]. Thus, based on this possibly dual facet of TNF-α, also observed in other cytokines and adipokines [25], we could speculate that the upregulation of TNF-α in NAFL and NASH may primarily target to a cytoprotective effect, when lipids are increasingly accumulated into the hepatocytes; however, if this hepatoprotective effect fails, TNF-α induces the cell death of the affected hepatocytes. Of course, this hypothesis remains to be shown.

TNF-α is also involved in the pathogenesis of hepatic fibrogenesis. TNF-α induces the production of transforming growth factor-beta (TGF-β) by the hepatocytes and KCs, which triggers the activation, differentiation, and proliferation of hepatic stellate cells (HSCs) [26]. In addition, TNF-α was shown to upregulate the expression of periostin in hepatocytes [27] and tissue inhibitor of metalloproteinase (TIMP)-1 in HSCs [28], which were suggested to facilitate collagen deposition and extracellular matrix stabilization, respectively. Other authors proposed that TNF-α enhances the survival of HSCs, but it may not be directly involved in the activation and differentiation of HSCs into myofibroblasts [29]; this may imply that TNF-α requires the contribution of other factors to exert a fully fibrogenic effect to the liver. Thus, though TNF-α appears to favor a fibrogenic response in the liver, the precise underlying mechanisms by which TNF-α affects HSCs and interplays with other fibrogenic contributors are not fully understood.

### Contribution of TNF-α to Insulin Resistance and NAFLD-Related Complications

TNF-α may link inflammation and IR, two hallmarks of NAFLD pathogenesis. Mice lacking TNF-α or TNFR were, at least partly, protected from IR in several “loss-of-function” experimental studies [30]. In line, hepatocyte-specific deficiency of TNFR1 protected mice from IR, but not NASH, as TNFR1 was deleted only in the hepatocytes, but not in KCs [31]. Furthermore, TNF-α administration was shown to induce IR, whereas inhibition of ΤNF-α was associated with improved insulin sensitivity [7]. Indeed, TNF-α may block insulin signaling in the hepatocytes at the post-receptor level; it triggers the expression of the suppressor of cytokine signaling (SOCS) proteins, which prevents tyrosine phosphorylation of insulin receptor substrates (IRS)-1 and IRS-2 and promotes their early degradation [32, 33]. Likewise, TNF-α-mediated activation of JNK contributes to the inhibition of insulin signaling (i.e., IR) through serine phosphorylation of IRS-1 and IRS-2 [24]. Moreover, TNF-α-induced SREBP-1c, in addition to promoting lipogenesis, also suppresses the IRS-1/2 synthesis, thus contributing to IR [24]. Notably, TNF-α also antagonizes adiponectin in NAFLD and suppresses its insulin-sensitizing effect on the hepatocytes [5, 8].

Therefore, TNF-α may integrate both inflammatory and metabolic signals, potentially connecting inflammation with IR, which synergically deteriorate NAFLD. Of note, these interconnecting mechanisms may also predispose to the development of NAFLD-related comorbidities, including CVD and HCC. Both IR and increased circulating TNF-α have been associated with NAFLD and CVD. Based on mechanistic studies, TNF-α may be one of the factors linking NAFLD, IR, and CVD [34, 35], the latter being the leading cause of mortality in patients with NAFLD [36]. In addition, we have recently focused on the interplay between inflammation, IR, and hepatic carcinogenesis, highlighting that IR, inflammation, and carcinogenesis in NAFLD may move hand-in-hand both intra- and extra-hepatically [37]. It has been supported that IR may directly be associated with HCC, independently from the classical inflammation-fibrosis cascade, by initiating intracellular mitogenic and anti-apoptotic pathways [38]. In this regard, TNF-α may be one of the mediators of this triadic interplay. TNF-α promotes hepatocellular tumorigenesis through the activation of hepatic progenitor cells [39]. Moreover, TNF-α-induces JNK, regarded as an oncogenic transcription factor, which is commonly activated in HCC [40].

**Fig. 1:**
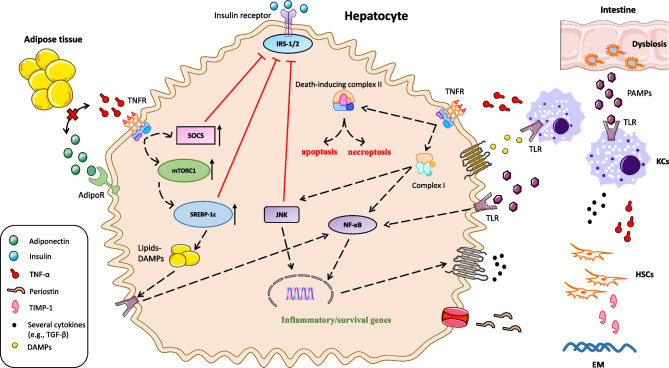
The role of TNF-α in the pathogenesis of NAFLD. In the liver, TNF-α is primarily produced by the KCs, which respond to two types of stimuli: the DAMPs, which are released by lipid-infiltrated and damaged hepatocytes, and the gut-derived PAMPs, which translocate from the intestine to the liver, due to an impaired intestinal epithelial barrier. Both DAMPs and PAMPs bind TLRs on the surface of the KCs and activate the NF-κB, which is the key signaling pathway for the transcription of TNF-α. Furthermore, fat-stressed hepatocytes also contribute to TNF-α production via the interaction between DAMPs/PAMPs and TLRs, albeit to a lesser extent. Once released, TNF-α binds TNFR and stimulates the assembly of complex I, which initiates two important downstream signaling pathways, i.e., the JNK and NF-κB pathways, through which TNF-α induces the transcription of target genes involved in inflammation, cell proliferation, and survival. Besides these cellular responses, TNF-α also induces cell death by enabling the assembly of the death-inducing protein complex II, leading to either apoptosis or necroptosis. Besides, inflammation, survival, and apoptosis, TNF-α also contributes to IR and NAFL development; TNF-α perpetuates IR in hepatocytes as it blocks insulin signaling at the post-receptor level; TNF-α triggers the expression of SOCS, which prevent tyrosine phosphorylation of IRS-1 and IRS-2 and promote their early degradation. Likewise, TNF-α-mediated activation of JNK contributes to the phosphorylation of serine of IRS-1 and IRS-2, which inhibits their signaling. In addition, TNF-α is proposed as a potential positive regulator of the mTORC1 pathway in the hepatocytes, inducing the expression of the SREBP-1c, the main transcription factor of *de novo* lipogenesis. TNF-α-induced SREBP-1c, in addition to promoting *de novo* lipogenesis, also suppresses the IRS-1/2 synthesis, thus contributing to IR. Notably, TNF-α also antagonizes adiponectin and suppresses its insulin-sensitizing effect on the hepatocytes. Finally, TNF-α is involved in hepatic fibrogenesis; TNF-α induces the production of TGF-β by the hepatocytes and KCs, upregulates the expression of periostin in the hepatocytes and TIMP-1 in HSCs, which facilitate collagen deposition and EM stabilization

The above considering, TNF-α seems to be an important cytokine that potentiates hepatic IR and exhibits potential steatogenic, inflammatory, fibrogenic, and apoptotic effects in the liver. As a result, increased circulating and hepatic TNF-α may be associated with the onset and progression of NAFLD and its connection with hepatic complications and extrahepatic comorbidities. Figure 1 illustrates the potential molecular mechanisms through which TNF-α is possibly implicated in the pathogenesis of NAFLD.

## TNF-α in NAFLD: Evidence from Clinical Studies

Although most experimental and mechanistic studies suggest a causal role of TNF-α in the onset and progression of NAFLD from the early to advanced disease, clinical observational studies on the association between circulating TNF-α and NAFLD have yielded contradictory results. Most studies suggested that NAFLD patients had higher levels of circulating TNF-α than controls [41–43]. However, some studies did not show significant difference in circulating TNF-α concentrations between individuals with and without NAFLD [44, 45]. Of note, among studies with histologic confirmation of NAFLD, some found higher plasma TNF-α levels in NASH compared with NAFL patients [46, 47], while other studies showed no difference in circulating TNF-α between NAFL and NASH patients [48, 49].

Because of these heterogeneous and inconsistent findings, two meta-analyses on this topic have attempted to quantify the results of existing observational studies. In a meta-analysis of 56 studies, we demonstrated that circulating TNF-α was associated with the severity of NAFLD: TNF-α was higher in patients with NAFL than in non-NAFLD controls and higher in patients with NASH than NAFL or controls [50]. Ιn meta-regression analysis, male ratio was positively associated with TNF-α in the comparison between patients with NASH and NAFL and could explain 36% of the heterogeneity in this comparison [50]; therefore, the male to female ratio may influence the association between TNF-α levels and NAFLD, which should be taken into account in the design of relevant future studies. In line, another meta-analysis also demonstrated marginally higher circulating TNF-α levels in patients with NAFLD than non-NAFLD controls (11 studies) [51]. In addition, higher circulating TNF-α levels in patients with than without NAFLD-associated fibrosis were also reported in this study [51]; however, this finding should be carefully interpreted, because only two studies were included in this subgroup comparison. Apart from cross-sectional and case–control studies, which inherently cannot prove causal relationships, a prospective cohort study reported that higher serum TNF-α levels at baseline in apparently healthy participants were associated with increased adjusted risk of developing NAFLD after 4 years of follow-up [52]. A selection of the relevant observational studies, mainly those with largest sample size (> 100 participants) are summarized in Table 1, whereas a more comprehensive summary has been reported in the above-mentioned meta-analyses [50, 51].

**Table 1:** Main characteristics and outcomes of selected clinical observational studies evaluating circulating TNF-α concentrations in patients with NAFLD.^*, a^

First author (year), origin [reference]	Study type	Population (*n*)	NAFLD definition	Method of TNF-α measurement	Main findings on circulating TNF-α	Additional information
Loguercio (2004), Italy [44]	Case–control	NAFL (61) NASH (237) NAFLD-cirrhosis (7)	Biopsy	ELISA	↔	-
Wong (2006), China [46]	Case–control	NAFL (28) NASH (52) Controls (41)	Biopsy	ELISA	NAFLD vs. controls ( ↔) NASH (↑) vs. NAFL	-
Kashyap (2009), USA [45]	Cross-sectional	NAFL (33) NASH (66) Controls (43)	Biopsy	ELISA	↔	Morbidly obese subjected to bariatric surgery
Koehler (2012), USA [48]	Case–control	NAFL (72) NASH + F 0–1 (60) NASH + F ≥ 2 (12) Controls (16)	Biopsy	ELISA	↔	Morbidly obese subjected to bariatric surgery
Seo (2013), South Korea [52]	Cohort	Apparently healthy individuals (non-NAFLD) at baseline (363)	US	ELISA	Higher baseline serum TNF-α was associated with increased risk of NAFLD (odds ratio: 2.20; 95%CI: 1.12–4.01)	106 participants developed NAFLD after 4 years of follow-up
Abdel-Razik (2016), Egypt [47]	Case–control	NAFL (753) NASH (120) Controls (150)	Biopsy	ELISA	NAFL (↑) vs. controls NASH (↑) vs. NAFL	Liver biopsy was performed in controls during cholecystectomy
Kapil (2016), India [43]	Case–control	NAFLD (60) CVH (32) Controls (50)	Biopsy	ELISA	NAFLD (↑) vs. controls NAFLD (↑) vs. CVH	-
Ajmera (2017), USA [49]	Cross-sectional	NAFL (143) Borderline NASH (129) Definite NASH (376)	Biopsy	ELISA	NAFL/borderline NASH vs definite NASH ( ↔) NAFLD + F0-1 (↓) vs NAFLD + F ≥ 2	-
Chellali (2019), Algeria [41]	Cross-sectional	T2DM (102) NAFLD (74) NAFLD + T2DM (54) Controls (90)	Biopsy	ELISA	NAFLD (↑) vs. controls NAFLD + T2DM (↑) vs. controls T2DM vs. controls ( ↔)	-
Federico (2019), Italy [42]	Baseline data from an interventional study	NAFLD (90) Controls (60)	Biopsy	ELISA	NAFLD (↑) vs. controls	Patients with GERD served as controls

↑, increase; ↓, decrease; ↔ , no difference between groups

*CVH* chronic viral hepatitis, *F* fibrosis, *GERD* gastroesophageal reflux disease, *NAFLD* nonalcoholic fatty liver disease, *NAFL* nonalcoholic fatty liver, *NASH* nonalcoholic steatohepatitis, *T2DM* type 2 diabetes mellitus, *TNF-α* tumor necrosis factor-α, *USA* United States of America, *US* ultrasound, *vs*. versus

^*^Data were derived from clinical observational studies with sample size of larger than 100 participants

^a^References are sorted according to the year of publication (primarily) and the surname of the first author (secondarily)

## Treatment Considerations

Due to the potential association between TNF-α and NAFLD, there are experimental and clinical studies targeting to evaluate the effect of various therapeutic interventions on TNF-α and NAFLD. Furthermore, anti-TNF-α therapies in various diseases have opened a possible therapeutic window for the management of advanced NAFLD.

### Pharmacologic Strategies Downregulating TNF-α in NAFLD

This section provides an overview of current and emerging pharmacologic strategies for NAFLD that reduce circulating or hepatic TNF-α based on prevailing experimental or clinical evidence. These data are also summarized in Tables 2 and 3.

**Table 2:** The effect of therapeutic interventions on TNF-α in experimental models of NASH.^*,a^

Intervention	Category	Experimental model	TNF-α measurement	TNF-α change
Obeticholic acid [54, 55]	FXR agonist	Male Ldlr − / − Leiden mice with HFD-induced NASH [54]; MC4R-KO mice with WD-induced NASH [55]	Hepatic TNF-α mRNA	↓
Obeticholic acid + Losartan [56]	FXR agonist + ARB	Male Fischer 344 rats with CDAAD-induced NASH	Hepatic TNF-α mRNA	↓
Obeticholic acid + Sitagliptin [57]	FXR agonist + DPP-4 inhibitor	Male Fischer 344 rats with CDAAD-induced NASH	Hepatic TNF-α protein	↓
Obeticholic acid + Simvastatin [58]	FXR agonist + statin	C57BL/6 J mice with HFD-induced NASH	Hepatic TNF-α mRNA	↓
Obeticholic acid + Elafibranor [59]	FXR agonist + PPAR-α/δ agonist	V-Lep ob/JRj (ob/ob) mice with HFD-induced NASH	Hepatic TNF-α mRNA	↓
Rosiglitazone [68, 69]	PPAR-γ agonist	Male Sprague–Dawley rats with HFD diet-induced NASH [68]; Wistar rats with MCDD-induced NASH [69]	Serum TNF-α	↓
Fenofibrate [72–74]	PPAR-α agonist	Male Sprague–Dawley rats with HFD-induced NASH [72]; male C57BL/6 mice with CDAHFD-induced NASH [73]; male Wistar rats with HFD-induced NASH [74]	Hepatic TNF-α mRNA [72, 74]; hepatic TNF-α protein [73]	↓
Saroglitazar [73–75]	PPAR-α/γ agonist	Male C57BL/6 mice with CDAHFD-induced NASH [73]; male Wistar rats with HFD-induced NASH [74]; DIAMOND mice with WD-induced NASH [75]	Hepatic TNF-α protein[73]; hepatic TNF-α mRNA[74, 75]	↓
Lanifibranor [77]	Pan-PPAR agonist	C57BL/6 mice with CDAHFD- and WD-induced NASH	Hepatic TNF-α mRNA	↓
Remogliflozin [80]	SGLT2 inhibitor	C57BL/6 J mice with HFD-induced NASH	Hepatic TNF-α mRNA	↓
Sitagliptin [57]	DPP-4 inhibitor	Male Fischer 344 rats with CDAAD-induced NASH	Hepatic TNF-α protein	↔
B1344 [95]	FGF-21 analog	C57BL/6 mice with MCDD-induced NASH	Hepatic TNF-α mRNA	↓
Rosuvastatin [98]	Statin	Male Sprague–Dawley rats with HFHCD-induced NASH	Hepatic TNF-α mRNA	↓
Simvastatin [58]	Statin	C57BL/6 J mice with HFD-induced NASH	Hepatic TNF-α mRNA	↓
Losartan [56]	ARB	Male Fischer 344 rats with CDAAD-induced NASH	Hepatic TNF-α mRNA	↔
Olmesartan [105]	ARB	Male Wistar rats with MCDD-induced NASH	Hepatic TNF-α mRNA	↓
Telmisartan [106]	ARB	Male Fischer 344 rats with CDAAD-induced NASH	Hepatic TNF-α protein and serum TNF-α	↓
Valsartan [104, 106]	ARB	Male Fischer 344 rats with CDAAD-induced NASH [106]; male Sprague–Dawley rats with MCDD-induced NASH [104]	Hepatic TNF-α protein [106] and serum TNF-α [104]	↓

↓, decrease; ↔ , no change

*ARB* angiotensin II receptor blockers, *CDAAD* choline-deficient L-amino-acid-defined diet, *DPP-4* dipeptidyl peptidase-4, *FGF-21* fibroblast growth factor-21, *FXR* farnesoid X receptor, *HFHCD* high-fat and high-cholesterol diet, *HFD* high-fat diet, *MCDD* methionine- and choline-deficient diet, *MC4R-KO* melanocortin 4 receptor-deficient, *NASH* nonalcoholic steatohepatitis, *PPAR* peroxisome proliferator-activated receptors, *SGLT2* sodium-glucose co-transporter 2, *TNF-α* tumor necrosis factor-α, *WD* western diet

^*^The table includes therapeutic agents with experimental, but without available clinical data, on their effect on TNF-α in NASH

^**a**^References are sorted according to the order mentioned in the text

**Table 3:** The effect of therapeutic interventions on TNF-α in patients with NAFLD as derived from clinical trials.^*,a^

Intervention (reference)	Category or action	Dosage	Type of study	Population (*n*)	Control	Duration	TNF-α measurement	TNF-α change	
Pioglitazone [66]	PPAR-γ agonist	30 mg od	Prospective cohort study	Biopsy-defined NASH (18)	-	12 months	Circulating TNF-α	↔	
Liraglutide [82]	GLP-1RA	1.8 mg od	RCT	T2DM + US-defined NAFLD (30)	Metformin	12 weeks	Circulating TNF-α	↓	
Probiotics [83]	Living microorganisms	Various	Meta-analysis of 21 RCTs	Biopsy- or MRS- or US-defined NAFLD (1037)	Placebo	NA	Circulating TNF-α	↔	
Synbiotics [84]	Synbiotics (Probiotics + Prebiotics)	Various	Meta-analysis of 7 RCTs	NAFLD (419)	Placebo	NA	Circulating TNF-α		↓
Rifaximin [87]	Antibiotic	550 mg bid	RCT	Biopsy-defined NASH (50)	Placebo	6 months	Circulating TNF-α	↓	
Vitamin E [89]	Antioxidant	300 mg bid δ-tocotrienol	RCT	US-defined NAFLD (100)	α-tocopherol	12 months	Circulating TNF-α	↓	
Pentoxifylline [90]	Phosphodiesterase inhibitor	Various	Meta-analysis of 3 RCTs and 2 prospective cohorts	Biopsy- or US-defined NAFLD (147)	Placebo, UDCA	Various	Circulating TNF-α	↓	
Pentoxifylline [91]	Phosphodiesterase inhibitor	Various	Meta-analysis of 5 RCTs	Biopsy- or US-defined NAFLD (157)	Placebo	3–12 months	Circulating TNF-α	↔	
UDCA + Vitamin E [93]	Bile acid + Antioxidant	12–15 mg/kg/day + 400 IU bid	RCT	Biopsy-defined NASH (41)	UDCA + placebo, placebo + placebo	24 months	Circulating TNF-α	↔	
Atorvastatin [97]	Statin	10 mg od	Prospective cohort study	Biopsy-defined NASH + dyslipidemia (42)	-	12 months	Circulating TNF-α	↓	
EPA [100]	n-3 PUFA	2700 mg od	Prospective cohort study	Biopsy-defined NASH (23)	-	12 months	Circulating TNF-α R	↓	
ALA/ EPA/ DHA + Diet [101]	n-3 PUFA + Diet	2 gr od + 30% caloric restriction	RCT	US-defined NAFLD (36)	Diet (30% caloric restriction)	6 months	Circulating TNF-α	↓	

Abbreviations: ↓, decrease; ↔ , no change, compared to control

*ALA* alpha-linolenic acid, *bid* twice daily, *CFU* colony-forming unit, *DHA* docosahexaenoic acid, *EPA* eicosapentaenoic acid, *GLP-1RA* glucagon-like peptide-1 receptor agonist, *MRS* magnetic resonance spectroscopy, *NA* not available, *NAFLD* nonalcoholic fatty liver disease, *NASH* nonalcoholic steatohepatitis, *n-3 PUFA* n-3 polyunsaturated fatty acids, *od* once daily, *PPAR* peroxisome proliferator-activated receptor, *R* receptor, *RCT* randomized-controlled trial, *T2DM* type 2 diabetes mellitus, *TNF-α* tumor necrosis factor-α, *tid* three times a day, *UDCA* ursodeoxycholic acid, *US* ultrasound

^*^The table includes selected meta-analyses, RCTs and prospective observational studies, considered to be the best available evidence to-date, according to the principles of evidence-based medicine

^**a**^ References are sorted according to the order mentioned in the text

Obeticholic acid (OCA) is a potent farnesoid X receptor (FXR) agonist, an intriguing class of medication under investigation for the treatment of NAFLD. In addition to controlling a number of metabolic processes, including bile acid synthesis, glucose homeostasis, and lipid metabolism, OCA also exhibits anti-inflammatory and anti-fibrotic properties in the liver [53]. OCA has been reported to reduce hepatic TNF-α expression, either as monotherapy [54, 55] or more potently when combined with other agents (e.g., simvastatin, losartan, sitagliptin, elafibranor) [56–59] in experimental animal models of NASH. In clinical trials, OCA improved histological features of NASH and, more importantly, fibrosis [60, 61], thus being an important candidate for the treatment of NASH, as long as safety concerns, including unfavorable effects on lipid profile and pruritus are addressed.

Peroxisome proliferator-activated receptors (PPARs) are a superfamily of lipid-sensoring nuclear receptors, which are considered promising therapeutic targets for NAFLD, as they regulate glucose and lipid metabolism, inflammation and possibly fibrosis [62]. Pioglitazone, a PPAR-γ agonist that belongs to thiazolidinediones, is a long-standing antidiabetic medication, usually preferred as a second- or third-line option [63]. Although pioglitazone was shown to reduce TNF-α experimentally [64], in human NASH, it improved hepatic steatosis, inflammation, and possibly marginally hepatic fibrosis [65], by increasing the levels of circulating adiponectin but without affecting circulating TNF-α [66]. Pioglitazone is currently recommended for *off-label* treatment in selected patients with biopsy-proven NASH and fibrosis stage (F) ≥ 2 [67]. Similarly, rosiglitazone, another PPAR-γ activator belonging to thiazolidinediones, also reduced circulating TNF-α in NASH rat models [68, 69]; however, no data regarding its effect on TNF-α in human NASH are available. In addition, rosiglitazone did not improve NASH in the phase II trial (FLIRT) [70]. It should be also noted that the use of rosiglitazone, initially approved as anti-diabetic medication, was restricted because of an increase in myocardial infarction risk [62]. Fenofibrate, a commonly used drug against hypertriglyceridemia with an agonistic effect on PPAR-α, has been investigated against NAFLD, due to its pleiotropic properties, including lipid-lowering and anti-inflammatory action [71]. In line, fenofibrate was shown to decrease hepatic TNF-α expression in mouse models of NASH [72–74]. Similar to rosiglitazone, fenofibrate effect on TNF-α in human NASH has not been investigated to-date. Saroglitazar, which is a dual PPAR-α/γ agonist, i.e., exerting combined effects on PPAR-α and PPAR-γ, reduced hepatic TNF-α expression and improved histology in experimental NASH models [73–75]. Saroglitazar is under evaluation in NASH patients with fibrosis, following encouraging results in a recent randomized controlled trial (RCT), in which saroglitazar successfully decreased liver function tests (LFTs) and liver fat content in NAFLD patients [76]. Lanifibranor, a pan-PPAR agonist, ameliorated all histological features of NASH in mice, including fibrosis, and reduced activation of macrophages and TNF-α expression mainly via PPAR-δ agonism [77]. Lanifibranor is another promising therapeutic candidate as it achieved both primary and secondary endpoints in human NAFLD (resolution of NASH and fibrosis), therefore is currently under investigation in a phase 3 RCT (NCT04849728). Of note, beyond pre-clinical studies, clinical evidence on the effect of dual-PPAR agonist (saroglitazar) and pan-PPAR agonist (lanifibranor) on TNF-α are scarce.

Following favorable effects on non-invasive biomarkers of hepatic steatosis and fibrosis by sodium-glucose co-transporter-2 (SGLT-2) inhibitors in patients with NAFLD [78], a phase 3 RCT with dapagliflozin is ongoing in patients with biopsy-proven NASH (NCT03723252). SGLT-2 inhibitors act primarily by inducing glucosuria; however, data from T2DM preclinical and clinical studies have also revealed potential anti-inflammatory action and reduction of some circulating cytokines, including TNF-α [79]. Clinical data on the effect of SGLT-2 on TNF-α in the setting of NAFLD are lacking. Experimentally, remogliflozin was found to reduce hepatic TNF-α mRNA in mice with diet-induced NASH [80]; nevertheless, additional evidence is required to establish a potentially anti-TNF-α effect of SGLT2 in NAFLD. Glucagon-like peptide-1 receptor agonists (GLP-1RAs) represent another class of anti-diabetic medication showing many favorable metabolic effects that make them an appealing therapeutic option for NASH. Semaglutide and liraglutide achieved higher rates of NASH resolution compared with placebo in phase 2 clinical trials, but without improving fibrosis [2]. Besides metabolic properties, liraglutide was shown to exert anti-inflammatory action through downregulating TNF-α in a mouse model of NASH [81], a finding that was further supported in an RCT of patients with concomitant T2DM and NAFLD, where liraglutide, as well as metformin, reduced circulating TNF-α [82]. However, more studies are needed to establish any anti-TNF-α potential of liraglutide and other GLP-1RAs. On the other hand, sitagliptin, a dipeptidyl peptidase-4 (DPP-4) inhibitor, an approved anti-diabetic medication, failed to decrease hepatic TNF-α in an experimental NASH rat model [57]; it is highlighted that DPP-4 inhibitors showed minimal or null effects on NAFLD [2].

A number of RCTs have evaluated the efficacy and safety of probiotics in the treatment of NAFLD. The rationale lies on the proposed ability of probiotics to modulate the gut microbiome, thus beneficially affecting the gut-liver axis. At present, various strains, preparations, dosage schemes, and durations of treatment have been investigated on different NAFLD-related endpoints, which increases the heterogeneity between studies, thus rendering hard any secure conclusions. Probiotics improved LFTs, hepatic steatosis, plasma glucose and insulin levels, and lipid profile, but they did not affect body mass index (BMI) or circulating TNF-α, according to a meta-analysis of 21 RCTs involving 1037 patients with NAFLD [83]. In contrast, synbiotic supplementation (i.e., nutritional supplements that combine probiotics and prebiotics), apart from improving LFTs, lipid profile, and glucose metabolism, had favorable effect on circulating TNF-α, in another meta-analysis of 7 RCTs including 419 NAFLD patients [84].

Rifaximin is a broad-spectrum minimally absorbable antibiotic, which targets dysbiosis of intestinal microbiota and related endotoxemia. A 6-week course of 800 mg rifaximin daily in 15 histologically-proven NASH patients was not associated with robust changes in LFTs, hepatic steatosis, insulin sensitivity, and pro-inflammatory serum cytokines, including TNF-α [85]. A previous 4-week trial using a higher dose of rifaximin (1200 mg) reduced lipopolysaccharides (LPS) and improved BMI and LFTs in 27 biopsy-proven NASH patients, but serum TNF-α concentrations remained unaffected [86]. The authors suggested that the higher dose of rifaximin is most likely to reduce endotoxemia, but the relatively short treatment period may probably be insufficient to suppress cytokines, including TNF-α. In accordance with this hypothesis, a longer (6-month) RCT in histologically-confirmed NASH patients showed that daily administration of 1100 mg rifaximin reduced circulating TNF-α along with LFTs, IR, and presumable hepatic steatosis. [87].

Vitamin E is a potent antioxidant, which may also exhibit anti-TNF-α action, as shown in rats with diet-induced NASH [88]. Indeed, the anti-inflammatory properties of vitamin E were also evidenced in an RCT of patients with ultrasound-defined NAFLD, where vitamin E, mainly in the form of δ-tocotrienol reduced inflammatory mediators, including TNF-α [89]. Of note, vitamin E at a daily dose of 800 IU resolves NASH, but not hepatic fibrosis, and may be prescribed *off-label* for selected NASH patients with F ≥ 2 for maximum 2 years [71].

Pentoxifylline is a xanthine derivative, which is a non-selective phosphodiesterase inhibitor, shown to histologically improve NASH in two meta-analyses [90, 91]. These meta-analyses, however, do not agree on the effect of pentoxifylline on circulating TNF-α; one of them, including 3 placebo-controlled RCTs and 2 ursodeoxycholic acid (UDCA)-controlled prospective cohort studies, reported that pentoxifylline decreases circulating TNF-α [90], while the latter, limited to RCTs (*n* = 5), did not show a significant difference in TNF-α levels between pentoxifylline and placebo [91]. Thus, more and probably more focused on TNF-α studies are needed to elucidate the potential effect of pentoxifylline on TNF-α.

UDCA, a secondary bile acid, was shown efficacy to reduce pro-inflammatory cytokines in animal studies [92]. Clinical trials have shown some effects of UDCA on LFTs and possibly hepatic steatosis, but minimal, if any effect on hepatic inflammation and fibrosis [71]. However, the combination of UDCA with vitamin E improved histology in patients with NASH, by increasing adiponectin levels and reducing hepatocellular apoptosis, but without affecting circulating TNF-α and other mediators of inflammation [93]. Currently, existing evidence does not support the use of UDCA in patients with NASH.

Fibroblast growth factor-21 (FGF-21) analogs or mimetics act at the same receptors as the endogenous hepatokine FGF-21, which is regarded as a promising molecule against hepatic steatosis, inflammation and apoptosis [94]. B1344, an FGF-21 analog, reduced the expression of TNF-α and other cytokines in the liver of mice with diet-induced NASH [95], corroborating earlier evidence showing FGF-21 analogs to be effective in both animal models and humans with NASH, although their direct effect on TNF-α has not been investigated in human NASH to-date [94]. As a result, numerous clinical trials with FGF-21 analogs are currently underway [94].

Atorvastatin, a strong and widely used statin for the treatment of dyslipidemia [96], was shown to improve lipid profile, LFTs, and NAFLD activity score (NAS) in an uncontrolled, interventional trial with 42 biopsy-confirmed NASH patients at a daily dose of 10 mg for 12 months; this effect was partly attributed to its lowering effect on circulating TNF-α [97]. Similarly, rosuvastatin and simvastatin both decreased hepatic TNF-α mRNA in experimental mice models of NASH, implying their potential anti-inflammatory properties on the liver [58, 98]. Overall, statin therapy appears to be safe in NAFLD patients and should be used at least to treat dyslipidemia and prevent cardiovascular events in patients with NAFLD, although their effects on hepatic histology are not well documented [96].

Due to their triglyceride lowering effect, N-3 polyunsaturated fatty acids (n-3 PUFAs) were considered to be beneficial for NAFLD [99]. Supplementation with eicosapentaenoic acid (EPA), one of the major components of n-3 PUFAs, or a mixture of EPA with docosahexaenoic (DHA) and alpha lipoic acid (ALA) plus caloric restriction, significantly reduced circulating TNF-α receptor or TNF-α, respectively, in NAFLD patients [100, 101]. However, RCTs with histological endpoints did not provide favorable results in NAFLD [102] and, therefore, n-3 PUFAs are not currently recommended for the treatment of NASH [71]. Different compositions and formulas used in research and in clinical practice further complicate secure conclusions on their effects on NAFLD.

Angiotensin receptor blockers (ARBs) have been proposed as alternative therapeutic option for NAFLD, mainly owing to their potentially anti-inflammatory and anti-fibrotic effects in the liver [103]. Both valsartan [104] and olmesartan [105] were reported to reduce hepatic TNF-α in experimental studies, and telmisartan was more effective than valsartan in reducing hepatic expression of TNF-α in rats with diet-induced NASH in a comparative study [106]. In contrast, losartan had no effect on the hepatic TNF-α mRNA in another experimental NASH rat model [56]. These variations may be attributed to structural differences between ARBs, as well as to telmisartan properties beyond angiotensin receptor, e.g., the activation of PPAR-γ [103]. The experimentally observed anti-TNF action of some of ARBs has not been demonstrated to-date in human NASH. However, an open-label prospective study with short-term administration of telmisartan to hypertensive patients with metabolic syndrome (MetS) showed an increase in circulating adiponectin and improvement in IR but no effect on serum TNF-α levels [107]; however, the results of this study could not directly be extrapolated to NAFLD patients.

### Anti-TNF-α Therapies: a Promising Therapeutic Approach for NAFLD Treatment

Given the potential steatotic, inflammatory, and fibrogenic effects of TNF-α in the liver, targeting TNF-α may be promising for the treatment of advanced NAFLD. TNF inhibitors are a class of biologic agents, which include infliximab, adalimumab, etanercept, golimumab, and certolizumab pegol, and have been widely used to treat chronic immune-related diseases, such as rheumatoid arthritis (RA), psoriatic arthritis (PsA), and inflammatory bowel diseases (IBD) [108]. Importantly, the extensive use of these agents in clinical practice over the last few decades has resulted in considerable experience on their efficacy and safety.

Experimental evidence has shown favorable effects of anti-TNF approaches on NAFLD outcomes; administration of infliximab in rats with diet-induced NASH resulted in histological regression of hepatic inflammation and fibrosis [109]**.** Hepatic steatosis, although improved, seems to be less affected by anti-TNF agents than other histological features of NAFLD [110].

In the clinical setting, initial reports on the metabolic and hepatic effects of anti-TNF biologics derive mostly from observational studies in patients with RA and PsA. PsA patients receiving a 24-month course of etanercept and adalimumab showed an improvement in metabolic syndrome components, like waist circumference, triglycerides, high-density lipoprotein-cholesterol (HDL-C), and glucose, when compared with those treated with methotrexate [111]. In line, other studies reported that anti-TNF reduced IR [112] and cardiovascular risk [113], both closely associated with NAFLD. Of note, administration of adalimumab to a young woman with rheumatoid arthritis and coexisting biopsy-proven NASH resulted in a remarkable biochemical response in terms of long-lasting improvements in LFTs [114]. Importantly, liver stiffness, which is associated with hepatic fibrosis, was lower in PsA patients treated with anti-TNF agents compared with those not on anti-TNF treatment, suggesting a possible antifibrotic effect [115]. On the contrary, some studies have raised the possibility that anti-TNF may increase body weight, which is a major risk factor for the development and progression of NAFLD [116]. There are also studies showing adverse hepatic effect after treatment with anti-TNF biologics. Administration of anti-TNF agents for 12 months in patients with PsA and US-defined NAFLD resulted in higher rates of worsening of hepatic steatosis compared with controls (with NAFLD but without PsA); the worsening of hepatic steatosis was greater in PsA patients with more active disease [117].

Concerning IBD populations, most studies showed higher prevalence of NAFLD in IBD patients than the general population [118]. Most studies do not reveal an association between biologic therapy and NAFLD incidence or severity [119]; however, existing studies are observational and were not designed towards this aim, i.e., the hepatic effect of biologics on NAFLD in IBD patients. It is highlighted that any possible benefit of anti-TNF inhibitors on NAFLD may be negated by a “rebound” weight gain observed following anti-TNF treatment, particularly in CD patients [108]. Anti-TNF medications may, in fact, ameliorate intestinal inflammation and achieve disease remission, which may lead to increased BMI and visceral adiposity due to restored nutrient absorption and increased appetite [108].

Clinical trials assessing anti-TNF medications specifically in NASH patients are not yet available. Before initiating such studies, observational studies with patients on anti-TNF agents for other conditions, who also have concomitant NASH, would be an excellent starting point [108]. Also, post hoc evaluations of existing clinical trials in patients with other diseases and concomitant NASH at baseline receiving anti-TNF agents may provide indirect insights into the effectiveness of anti-TNF agents on NASH. Positive outcomes in such observational studies may possibly support clinical trials with anti-TNF agents specifically in NASH, ideally with histological endpoints or non-invasive biomarkers of hepatic steatosis and fibrosis as acceptable alternatives [108]. Undoubtedly, we anticipate future studies designed to evaluate the role of anti-TNF-α agents in the treatment of NASH*.*

## Conclusions

Collectively, based on current mechanistic and experimental studies, TNF-α seems to be a contributor, critically implicated in the onset and progression of NAFLD. Furthermore, clinical data are showing higher circulating concentrations of TNF-α in NAFLD patients. Importantly, TNF-α may be associated with disease severity; more advanced phenotypes (i.e., NASH or NAFLD-related cirrhosis) seem to be associated with higher circulating TNF-α, which may also link NAFLD with extrahepatic manifestations, such as CVD and malignancies.

Based on these considerations, this topic may have valuable perspectives and clinical implications, but also considerable challenges. TNF-α has been suggested as a potentially useful biomarker for the non-invasive stratification of patients with NAFLD [120], an hypothesis, however, warranting diagnostic accuracy studies to be verified and possibly to provide specific cut-offs of TNF-α to rule out or rule in the diagnosis of NASH or NAFLD-associated fibrosis. In our opinion, the incorporation of TNF-α in combined diagnostic algorithms seems to be a more realistic potential rather the use of TNF-α alone. Therapeutically, a number of NAFLD pharmacotherapies appear to lower TNF-α, thus their effects may be mediated, at least in part, through suppressing TNF-α, which, however, remains to be elucidated. Interestingly, anti-TNF biologics have shown favorable metabolic and hepatic effects both in experimental models of NASH, as well as in observational studies involving patients with immune-related diseases, which may provide the rationale for repurposing ant-TNF agents in the treatment of NASH, thus opening a new therapeutic window for the management of advanced NAFLD. However, we should also bear in mind that TNF-α is a complex molecule with diverse functions, thus blocking its activity may have unintended consequences. In this regard, it has been reported that anti-TNF agents may increase the risk of serious infections, as well cancer risk [121]. In addition there are concerns about weight gain during treatment with anti-TNF medications, which should also be taken into account when treating patients with NAFLD, the majority of whom are obese. Thus, before proceeding with further research in the field, a balanced assessment of risk–benefit ratio is highly required. It seems that an individualized approach is needed in NAFLD patients, so different management may be needed for different patients [122]; in this aspect, anti-TNF medications may possibly be proven valuable for selected only NAFLD patient in the future.

## Data Availability

No datasets were generated or analyzed during the current review article.

## Abbreviations

AdipoR: Adiponectin receptor
DAMPs: Danger-associated molecular patterns
EM: Extracellular matrix
HSCs: Hepatic stellate cells
IR: Insulin resistance
IRS-1/2: Insulin receptor substrate-1/2
JNK: C-Jun N-terminal kinase
KCs: Kupffer cells
mTORC1: Mammalian target of rapamycin complex
NAFL: Nonalcoholic fatty liver
NF-κΒ: Nuclear factor-kappa B
PAMPs: Pathogen-associated molecular patterns
SOCS: Suppressor of cytokine signaling proteins
SREBP-1c: Sterol regulatory element-binding protein-1c
TGF-β: Transforming growth factor-beta
TIMP-1: Tissue inhibitor of metalloproteinase-1
TNF-α: Tumor necrosis factor-α
TNFR: TNF receptor
TLR: Toll-like receptor
